# A bioeconomic analysis of objective-based management options for late-stage emerald ash borer (Coleoptera: Buprestidae) infestations

**DOI:** 10.1093/jee/toaf037

**Published:** 2025-04-04

**Authors:** Alexander J F Martin, Lukas G Olson, Amory Ngan, Tenley M Conway

**Affiliations:** Department of Geography, Geomatics and Environment, University of Toronto Mississauga, Mississauga, ON, Canada; Department of Forest Resources Management, Faculty of Forestry, University of British Columbia, Vancouver, BC, Canada; Institute of Forestry & Conservation, John H Daniels Faculty of Architecture, Landscape & Design, University of Toronto, Toronto, ON, Canada; Parks, Forestry & Environment Division, Community Services Department, City of Mississauga, Mississauga, ON, Canada; Department of Geography, Geomatics and Environment, University of Toronto Mississauga, Mississauga, ON, Canada

**Keywords:** basal injection, chemical control methods, economic modeling, integrated pest management, invasive species management

## Abstract

Following its North American introduction, the emerald ash borer (*Agrilus planipennis* Fairmaire) (Coleoptera: Buprestidae) (EAB) has devastated ash populations (*Fraxinus* Linnaeus) (Oleaceae), largely extirpating the genus from infested regions. Previous cost-benefit analyses of EAB management options, including insecticidal injections, preemptive removals, and replanting, have examined early-stage infestations. This study tests options for late-stage EAB management based on ecological and economic objectives. We parameterized management decisions to evaluate tree counts, basal area, and urban forest value under 7 management options, varying if and when ash trees were injected, removed, and replanted with non-ash species. The simulation is applied to the remaining ash population in Mississauga, Ontario where tree coring and annual assessments determined that injected trees have reduced growth rates and are declining in condition. The results demonstrate that injections help preserve the ash population, maximize basal area, minimize spikes in annual costs, and reduce cumulative costs earlier in the 20-yr study period. However, long-term cost reduction is achieved through ceasing injections and removing ash as they die from EAB. Maintaining tree counts and maximizing net value is achieved through proactive replanting and winding down basal injections, coupled with a slow rate of removal, ultimately bringing the SLow Ash Mortality approach to a close.

## Introduction

The sustainability of the urban forest is important in maintaining the livability of cities because of the many ecosystem services they provide (O’Brien et al. 2022). As municipalities seek to effectively manage their urban forests with long-term sustainability in mind, the costs of maintaining or replacing trees, alongside the value of specific services, must be considered (Vogt et al. 2015). One urban forest management challenge facing many urban areas is the legacy of historical planting practices that often opted for streets of uniform species due to aesthetic preferences (Roman et al. 2018, Roman and Eisenman 2022). This resulted in low diversity urban forests that are vulnerable to insects and pathogens (Lacan and McBride 2008). Such vulnerability became particularly apparent in North America with the arrival of Dutch elm disease (*Ophiostomaulmi* (Buisman) Melin & Nannf. (1934)) (Ophiostomatales: Ophiostomataceae) in the mid-20th century and, more recently, emerald ash borer (EAB) (*Agrilus planipennis* Fairmaire (1888)) (Coleoptera: Buprestidae). When EAB arrived in North America in the 1990s, it spread rapidly across Canada and the United States, extirpating ash (*Fraxinus* Linnaeus) (Oleaceae) in affected areas (Herms and McCullough 2014). Urban foresters responded with several management tactics, including preemptive removals and the injection of insecticides into the base of the tree (McCullough 2020). Given that these are costly methods (McKenney et al. 2012, Hauer and Peterson 2017), it is important to compare their efficacy and economic impact. Several research studies concern the latter (McCullough and Mercader 2012, Vannatta et al. 2012, Kovacs et al. 2014, Sadof et al. 2017); however, all of these studies examine early-stage EAB, beginning the simulations before, immediately upon, or shortly after the arrival of EAB. In this study, we seek to resolve this gap by conducting a bioeconomic analysis of EAB management tactics tied to current common urban forestry objectives for late-stage infestations. Data from Mississauga, Ontario is used to parameterize the model. By using Canadian datasets and parameterizing declining tree conditions over time, we demonstrate the applicability of the model for communities managing remaining ash trees two decades after the initial infestation.

## Emerald Ash Borer and its Management

Native to northeastern Asia, EAB is an invasive buprestid that arrived in North America on infested wood packing materials in the 1990s (Aukema et al. 2011, Siegert et al. 2014), although its first detection only occurred in 2002 in Windsor, Ontario and southeast Michigan (Thordarson 2020). It has since spread across Canada and the United States, ranging from the eastern seaboard to, in Canada, Winnipeg, Manitoba in 2017 (MacDonald et al. 2022).

Its rampageous movement across North America has proven highly destructive. Following its detection, ash population mortality can exceed 99% within 8 to 10 yr (Klooster et al. 2014). Ultimately, ash may be completely extirpated from infested areas (Herms and McCullough 2014, Duan et al. 2023). The harm to ash occurs primarily during the larval stage. After the EAB larvae hatch, they chew through the bark and into the inner phloem, cambium, and outer xylem (Cappaert et al. 2005). As the larvae increase in size, the width of the galleries expand, resulting in unrecoverable damage to the tree (Chamorro et al. 2012). The larvae then overwinter in the sapwood or outer bark before pupation occurs in spring (Crosthwaite et al. 2011, Sobek-Swant et al. 2012). The adult beetle emerges from the infested ash, flying to feed on the leaves of nearby ash trees before mating (Cappaert et al. 2005, Wang et al. 2010b). However, the resulting defoliation by the adult beetle is not nearly as consequential to tree mortality as is the larval feeding damage (Herms and McCullough 2014).

The EAB life cycle is often annual, although it may be biannual with the occurrence of late oviposition or low larval densities (Cappaert et al. 2005, Siegert et al. 2010, Tluczek et al. 2011). If a biennial lifecycle occurs, the early-instar larvae will overwinter in the sapwood, continue developing the following summer, and overwinter the second year as prepupae before emerging as adults the following spring (Tluczek et al. 2011). While annual versus biennial lifecycles have thus far not been found to be associated with either climate or latitudes in North America (Tluczek et al., 2011), the differences will ultimately influence population growth rates (MacDonald et al. 2022).

Despite the non-association of climate and latitude with EAB lifecycles in North America (Tluczek et al. 2011), ash mortality rates decrease in colder regions due to delayed EAB emergence and peak activity dates (MacDonald et al. 2022). In regions like the Canadian prairies—the western EAB frontier—the cold climate limits the suitability of the region for EAB, mitigating its impacts (DeSantis et al. 2013, Lyons 2015, Barker et al. 2023). Climate is therefore considered the principal mitigator in the spread of EAB.

Climate, however, cannot be controlled for the benefit of EAB management. Consequently, several biological controls have been developed. Woodpeckers are a commonly referenced predator of EAB, although the predation is not sufficient to minimize large-scale mortality (Lindell et al. 2008, Duan et al. 2010). Parasitoid wasps (eg *Spathius agrili* Yang*, Tetrastichus planipennisi* Yang*, Oobius agrili* Zhang and Huang) and the fungal pathogen *Beauveria bassiana* (Bals.-Criv.) Vuill. have seen only limited implementation (Castrillo et al. 2010, Wang et al. 2010a, Duan et al. 2011, Yang et al. 2012, Srei et al. 2020).

At present, the common EAB management techniques are insecticidal and mechanical controls. Under the Slow Ash Mortality (SLAM) approach (McCullough and Mercader 2012, Herms and McCullough 2014), ash trees receive proactive microinjections of insecticide, largely considered the most economical approach to EAB management given the ecosystem services contributed by mature ash trees and the cost of tree removal (Vannatta et al. 2012). As a component of SLAM, early detection programs are conducted using sampling and pheromone trapping (McCullough and Mercader 2012). Once EAB is detected, the area is quarantined and the movement of ash is regulated (Hope et al. 2021). Trees found to be infested with EAB are rapidly removed to impede EAB population growth. The costly nature of these programs frequently limits replacement tree planting budgets (Hauer and Peterson 2017) and, as a result, areas impacted by EAB often go without replanting for several years.

### Modeling of EAB

Ecological modeling is an important method for studying the results of management inputs on ecological systems (Jackson et al. 2000). Inherently, models are simplifications of real ecological systems meant to improve understanding, aid conceptualization, and predict consequences of environmental decision making (Hall and Day 1977). Ecological modeling in urban forestry has included simulating EAB (Anderson and Dragićević 2015), Indianmeal moth (*Plodia interpunctella* Hübner (1813)) (Fontenot et al. 2013), and Dutch elm disease (Bajeux et al. 2020). These applications of urban forest modeling can be grouped into two broad types: growth models and economic models.

Pathogenic population or growth models simulate the spread of an insect or pathogen through the urban forest, often using multi-agent modeling. In EAB modeling, spatial dynamic and agent-based modeling have been used to simulate infestation dynamics and spread patterns (BenDor et al. 2006, Muirhead et al. 2006, Anderson and Dragićević 2015, Siegert et al. 2015). Related to spread patterns, Cuddington et al. (2018) proposed a mechanistic model to determine the EAB risk to cities based on overwintering mortality estimates. These growth models were limited in their estimates of economic impacts.

Economic models of EAB primarily focus on the costs of infestation, exploring either cost projections or cost-benefit analyses. For example, Kovacs et al. (2010) used a cost projection approach to estimate an EAB management cost of $10.7 billion USD in 25 US states, although this cost can vary depending on the number and expansion rate of satellite populations (Kovacs et al. 2011). At a finer scale, Vannatta et al. (2012) used a cost-benefit analysis to examine how different treatment options influenced urban forest value. Similar cost projection models and cost-benefit analyses have been constructed for the Canadian context where treatment options are more limited, including at the household scale (McKenney and Pedlar 2012), city scale (McKenney et al. 2012), and nation-wide (Hope et al. 2021).

While these previous economic models have used different modeling approaches and geographies, they are all focused on pre- or early-stage infestation, rather than late-stage infestations where few ash trees remain. To assist urban foresters in managing late-stage infestations, we constructed a model that considers the management of remnant ash populations. We test the model for common urban forestry objectives, including (i) tree (stem) maximization, (ii) basal area maximization, (iii) expense minimization, and (iv) value maximization. This differs from previous EAB models that frame their conclusions based on the assumption that value maximization is the primary objective. Our model also includes Canadian ash growth and mortality rates given the different insecticidal options available on the Canadian market. For urban foresters, our results will help guide the management of remnant ash populations amidst the conclusion of EAB management programs. For entomologists and urban ecologists, our results demonstrate the need to tie model results to practical objectives as examining different objectives can alter the conclusions and recommendations made by researchers.

## Methods

### Study City

Mississauga (43.5953°N, 79.6406°W, 156 m above sea level) is the seventh most populous municipality in Canada and the third most populous in Ontario. It has a population of 717,961 people at a density of 2,452.5 people per km^2^ (Statistics Canada 2022). Temperatures in Mississauga range from a monthly average of −5.0 °C in January to 22.1 °C in July (Government of Canada 2024). The mean annual precipitation is 806.8 mm, predominantly occurring as rainfall (697.4 mm) (Government of Canada 2024). Mississauga is in Canadian plant hardiness zone 6b (Natural Resources Canada 2022) and has a canopy cover of 15% (City of Mississauga, 2014). An estimated 2.1 million trees comprise the city’s urban forest across its land area of 292.74 km^2^, predominantly comprised of Norway maple (*Acer platanoides* L.), green ash (*Fraxinus pennsylvanica* Marshall), and honey locust (*Gleditsia triacanthos* L.). Mississauga’s urban forest is an important part of the city's identity, and urban trees are recognized as important to residents.

The Forestry division of the City of Mississauga is mandated to protect, enhance, restore, expand, and connect Mississauga’s urban forest and natural heritage system (City of Mississauga 2024b), which is comprised of natural assets (trees, natural areas, and boulevard landscapes) with a replacement value of over $931 million (City of Mississauga 2024c). This includes close to 300,000 street trees and over one million trees in parks and natural areas (City of Mississauga 2024b). To fulfill its mandate, the Forestry division plans and delivers a broad range of services such as tree maintenance, tree planting, tree protection, tree permits, bylaw enforcement, development and design review, forest management, naturalization, invasive species management, habitat restoration, stewardship and education, and boulevard vegetation management (City of Mississauga 2024b).

EAB was first detected in Mississauga in 2008. At the time, close to 10% of the 2.1 million public and private trees in Mississauga were ash trees. City Council adopted an EAB Management Plan in 2012, funded by a Special Purpose Levy of approximately $51 million over a 10 yr period, to treat viable City-owned ash trees on streets and parks and to remove and replace dead or dying ash trees that could not be retained (City of Mississauga 2014). As part of this plan, approximately 20,000 City-owned ash trees were initially identified for treatment with TreeAzin. Today, 1,490 City-owned ash trees remain.

### Tree and Maintenance Parameters

Tree parameters were calculated based on Mississauga’s tree inventory (Table 1), unless otherwise indicated. To incorporate management costs into the model, we used the 2024 staff and contractor costs and rates from the City of Mississauga’s competitive bid procurement records (Table 2). Costs were provided based on the City of Mississauga’s 2024 Fees & Charges Bylaw in accordance with Section 391 of the *Municipal Act*, 2001.

**Table 1.** Tree and maintenance parameters used in the models. Unless otherwise indicated in the Description, the value is provided by the City of Mississauga. All economic values are provided based on 2024 costs.

Parameter	Value	Description
Natural Mortality Rate	0.85%	Natural (“background”) mortality rate across all tree species per year.
Injected Ash Mortality Rate	2.8%	Mortality rate of ash tree population while being injected with TreeAzin.
Non-Injected Ash Mortality Rate	20%	For non-injected ash trees, the annual mortality rate is set at 20% per year (Vannatta et al. 2012).
Ash Tree Growth Rate (cm/yr)	0.3	Annual growth rate of diameter at breast height in cm for ash trees.
Non-Ash Tree Growth Rate (cm/yr)	0.47	Annual growth rate of diameter at breast height in cm for non-ash trees (Fleming and Steenberg 2023).
Tree planting and establishment	$849.91	Cost to plant and establish a 6 cm caliper tree.
Ash tree injections ($ / cm)	$3.325	Cost of TreeAzin injections per cm diameter at breast height.
Annual Inflation Rate	2%	Per the Bank of Canada (2024).
Annual Discounting Rate	2.83%	Per the City of Mississauga (2024a).

**Table 2.** Cost of pruning and removal of public trees in the City of Mississauga per diameter at breast height (DBH) based on the competitive bid procurement data.

DBH range (cm)	Removal cost ($)	Pruning costs
0–20	105	60
> 20–40	305	118
> 40–60	855	268
> 60–80	1,450	460
> 80–100	2,950	610
>100–120	2,950	710
> 120	5,700	862

We used the internal tree survivability statistics, available for the first 4 yr after a tree is planted, at the City of Mississauga to determine tree survival after planting (Table 3). We determined costs based on the warranty period. If the tree dies within 2 yr after planting, it is replaced free of charge under the contractor warranty. Starting at 3 yr, the tree is post-warranty and the city is responsible for replanting costs, a cost that is charged within the model. At 5 yr, the model reverts to the normal tree mortality rate of 0.85%, which is based on the City of Mississauga’s internal data.

**Table 3.** Annual survival rates for the first 4 yr post-planting based on 2020 to 2024 data provided by the City of Mississauga.

Years since planting	Warranty stage	Alive (%)	Dead (%)
1	Warranty	91.5	8.5
2	Warranty	88.3	11.7
3	Post-Warranty	95.8	4.2
4	Post-Warranty	93.9	6.1

### Tree Value

The tree appraisal method used in the model was developed by the Council of Tree & Landscape Appraisers (2019) (CTLA) and is widely recognized in Canada. Using the CTLA method, the value of the mature tree is related to the number of replacement trees required to achieve the same trunk area. The value of the mature tree is discounted by the tree’s condition, location rating, and species rating.

Based on the Mississauga inventory data, we set the ash tree condition rating at 50%, non-ash condition rating at 75%, and functional limitations rating at 75%. We set non-ash external limitations ratings at 90% while the external limitations rating for ash was set at 10% per CTLA, which recommends a 10% rating when a lethal pest is present in the area of the tree (Council of Tree & Landscape Appraisers 2019). While tree conditions ratings are individually assigned in tree inventories, we used median condition ratings to reduce computational intensity per Vannatta et al. (2012). We used a cost of $4.50 per cm^2^ for the purchase of a new tree. The CTLA value is given as


$$\begin{array}{l} CTLAValue=(DBH/2{)}^{2}*Pi*NT*(TC*FL*EL) \end{array}$$ (1)

where DBH is the diameter at breast height (cm) of the existing tree, NT is the cost of a new tree per cm^2^, TC is the tree condition (%), FL is the functional limitations rating (%), and EL is the external limitations rating (%).

We apply an inflation rate of 2% per annum to estimate the future value of the tree, the midpoint of the inflation-control target of the Bank of Canada (2024). The future value is presented per McKenney et al. (2012) and Brukas et al. (2001) with a 0% discount rate to show the impact of discounting. Figures and tables for the future values are provided in the Supplementary Material. To convert future value to present value, we applied a discount rate of 2.83% per annum based on the City of Mississauga’s discount rate for long-term assets (City of Mississauga 2024a). This is higher than the 2% discount rate used by Vannatta et al. (2012) and Kovacs et al. (2010) but lower than the 3%, 4%, and 5% discounting rates used in industrial/commercial forestry (Price 2014).

### Costs: Management and Scenarios

The management costs are comprised of (i) tree replacement and establishment, (ii) pruning, (iii) injections, (iv) removals, and (v) stumping. Several rules in the model are established for each of these management components. For tree planting, trees are replaced at a 1:1 ratio. Unless otherwise specified for the scenario, tree planting can occur before removal when alternative planting locations are selected. Dead ash trees are replaced with non-ash trees. Pruning occurs on a 4-yr cycle, with a quarter of the tree population pruned each year, and injections occur on a 2-yr cycle, with half of the tree population injected each year. Removals and stumping occur in the same year as mortality.

We ran the model for 7 different scenarios: (i) remove only (control); (ii) remove then replant; (iii) preemptive removal then replant; (iv) replant, inject, then preemptive removal; (v) inject, preemptive removal, and replant; (vi) injection in perpetuity; and (vii) injection in perpetuity with replanting. These scenarios are further described in Table 4.

**Table 4.** Descriptions of the 7 scenarios used in the models.

Scenario	Description
Remove Dead Ash Only (Control)	Ash trees are no longer injected. The remaining ash trees die due to emerald ash borer infestation at a rate of 20% per year and are removed after dying.
Remove Dead Ash then Replant	Follows Remove Dead Ash Only (Control) scenario but adds replanting. Trees are replanted in the same year.
Preemptive Removal then Replant	Ash trees are removed preemptively at a rate of 400 trees per year then replanted in the same year at a rate of 400 trees per year.
Replant, Inject, then Preemptive Removal	Replacement trees are planted at alternative sites at a rate of 400 trees per year beginning in Year 1. The remaining ash trees are injected for 5 yr before being removed at a rate of 400 trees per year.
Inject, Preemptive Removal, and Replant	Ash trees are injected for 5 yr then removed at a rate of 400 trees per year. The trees are replaced in the same year at a rate of 400 trees per year.
Injection in Perpetuity	The remaining ash trees continue to be injected for the duration of the 20-yr period. Only dead ash trees are removed.
Injection in Perpetuity with Replanting	The remaining ash trees continue to be injected for the duration of the 20-yr period. Only dead ash trees are removed. For every removed ash tree, a non-ash tree is replanted in the same year.

### Model Implementation and Objectives

To conduct the cost-benefit analysis, we used a population-level model with median data from the Mississauga tree inventory. The model is run for annual timesteps across a 20-yr period. For each timestep, trees grow in DBH per Table 1. We set both the removal rate and planting rate at 400 trees per year. Trees were injected from Year 1 to Year 5, and the removal year began in Year 5. Planting started in Year 1. These conditions best reflect the current management possibilities for the City of Mississauga.

The previous cost-benefit analyses of EAB have assumed that value maximization is the primary objective. We propose alternative objectives upon which we analyze our model. These objectives are:

1. **Preserve the existing number of trees.** This objective prefers management options that will ensure that the number of trees (stem count) is retained (Fleming and Steenberg 2023) at the lowest cost. This is based on common urban forestry planting and retention targets based on tree counts.
2. **Preserve the basal area**. This objective prefers management options that will ensure basal area, calculated from DBH, is retained at the lowest cost. This is based on the correlation between ecosystem services (value) and basal area.
3. **Preserve the ash population**. This objective prefers management options that will retain the ash population, regardless of cost. This objective is based on diversity management and biological preservation of the genus.
4. **Cost reduction**. This objective prefers management options that will result in the lowest net cost over a 20-yr period. This objective is based on the conclusion of leveraged EAB management funds that were used to support municipal response to EAB.
5. **Value maximization**. This objective prefers management options that will maximize value, calculated using the CTLA formula minus the management costs. This objective is based on asset management principles proposed in previous EAB analyses.

Our model is analyzed based on these above objectives. For each objective, we report the best management option to maximize these objectives. These results form the basis of our recommendations, which we elaborate on in the Discussion.

### Modeling Environment, Tradeoffs, and Sensitivity Analysis

The simulation code was written and run in PyCharm (2024.2.2) (JetBrains; Prague, Czech Republic), an integrated development environment for the programming language Python (3.12) (Python Foundation). The simulation code is available at https://github.com/arbmarta/Emerald-Ash-Borer-Management-Model.

We considered tradeoffs based on the discounted annual cost per tree and per 1,000 m^3^ of basal area. This helped to normalize the cost comparison to better compare between the management techniques. We also considered the ratio of discounted annual and cumulative costs to the Remove Dead Ash Only management option, which we treated as the control.

In addition to the conditions used in our simulation, we assessed how changes to management rates and tree variables influence the model outputs. Firstly, the optimization of different management decisions depends on 4 management decisions, namely (i) the year that injections are ended, (ii) the year that removals start, (iii) the rate of tree removals per year, and (iv) the number of new trees planted per year. These 4 decisions are included in at least one of 3 management options: Preemptive Removal then Replant (iii); Replant, Inject, the Preemptive Removal (ii, iii, & iv); and Inject, Preemptive Removal, and Replant (i & iii). We varied the year that injections end and the year that removals start between 1 and 20 yr. We varied both the rate of tree removed per year and trees planted per year between 100 and 1000 trees.

## Results

**Fig. 1.**
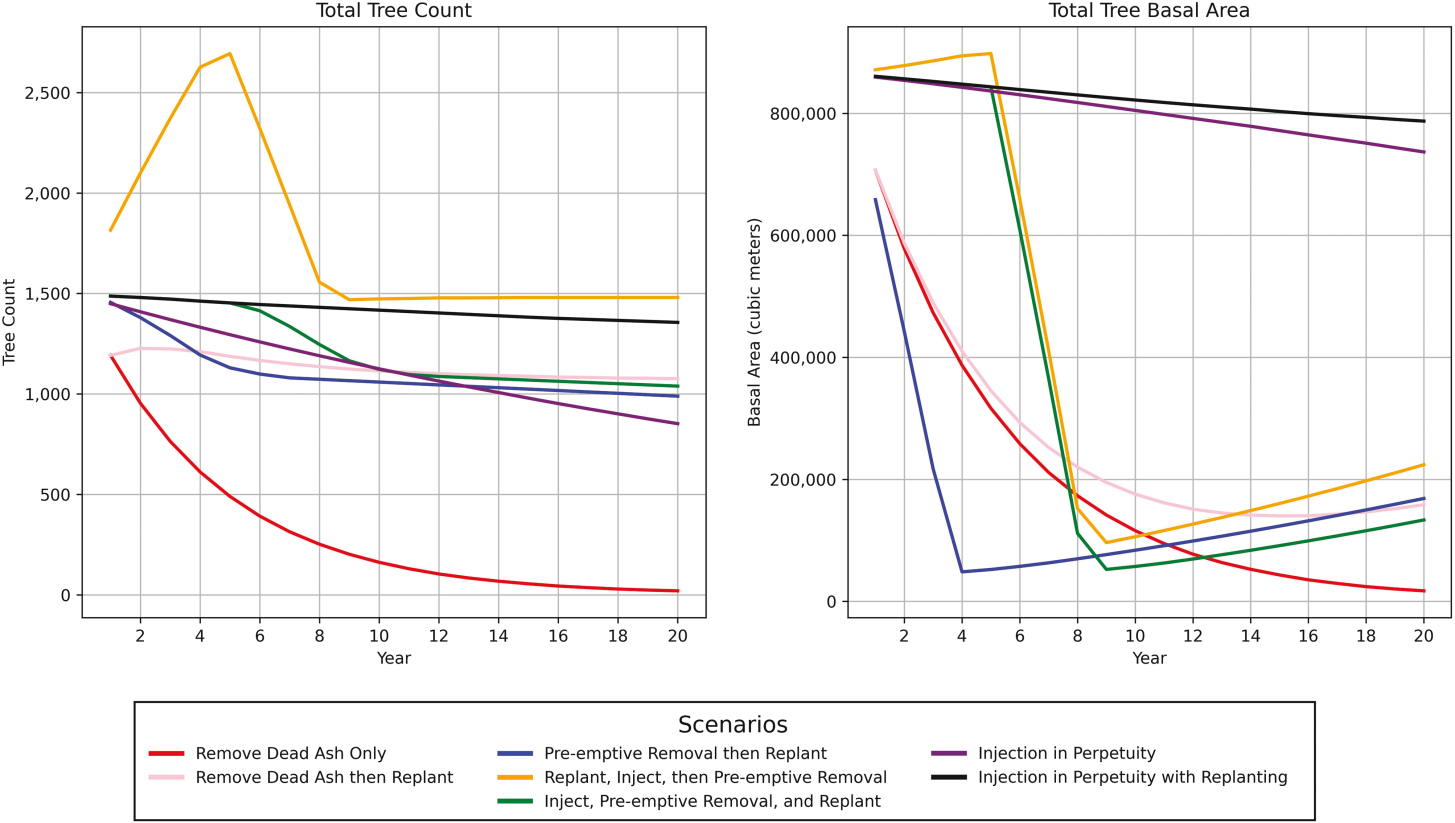
Tree count and basal area under 7 EAB management scenarios.

An overview of tree count and basal area over the 20-yr period is shown in Fig. 1. At Year 20, minor remnant ash populations remained under the Remove Only and Remove then Replant options (Fig. 1, Table 5) while the two management options with continued injections retained 852 ash—equal to 57.18% of the ash population in Year 0 (*N* = 1490)—and had the highest basal area (m^3^). The total tree count was highest for Inject in Perpetuity with Replanting, followed by Replant, Inject, then Preemptive Removal. The Remove Only option resulted in the fewest remaining trees (*n* = 20), followed by Injection in Perpetuity (*n* = 852).

**Table 5.** Tree count and basal area statistics of 7 emerald ash borer management options at Years 1, 5, 10, 15, and 20.

Metric	Tree species	EAB management scenario	Year 1	Year 5	Year 10	Year 15	Year 20
**Tree Count**	**Ash Trees**	Remove Dead Ash Only	1192	490	162	55	20
		Remove Dead Ash then Replant	1192	490	162	55	20
		Preemptive Removal then Replant	1090	0	0	0	0
		Replant, Inject, then Preemptive Removal	1449	1295	0	0	0
		Inject, Preemptive Removal, and Replant	1449	1295	0	0	0
		Injection in Perpetuity	1449	1295	1125	979	852
		Injection in Perpetuity with Replanting	1449	1295	1125	979	852
	**Non-Ash Trees**	Remove Dead Ash Only	0	0	0	0	0
		Remove Dead Ash then Replant	0	697	953	1033	1056
		Preemptive Removal then Replant	366	1130	1059	1024	989
		Replant, Inject, then Preemptive Removal	366	1399	1473	1480	1480
		Inject, Preemptive Removal, and Replant	38	158	1122	1069	1039
		Injection in Perpetuity	0	0	0	0	0
		Injection in Perpetuity with Replanting	38	158	292	403	504
	**All Trees**	Remove Dead Ash Only	1192	490	162	55	20
		Remove Dead Ash then Replant	1192	1187	1115	1088	1076
		Preemptive Removal then Replant	1456	1130	1059	1024	989
		Replant, Inject, then Preemptive Removal	1815	2694	1473	1480	1480
		Inject, Preemptive Removal, and Replant	1487	1453	1122	1069	1039
		Injection in Perpetuity	1449	1295	1125	979	852
		Injection in Perpetuity with Replanting	1487	1453	1417	1382	1356
**Basal Area** (per 1000 m^3^)	**Ash Trees**	Remove Dead Ash Only	707.0	316.6	115.9	43.4	17.3
		Remove Dead Ash then Replant	707.0	316.6	115.9	43.4	17.3
		Preemptive Removal then Replant	646.5	0.0	0.0	0.0	0.0
		Replant, Inject, then Preemptive Removal	859.4	836.6	0.0	0.0	0.0
		Inject, Preemptive Removal, and Replant	859.4	836.6	0.0	0.0	0.0
		Injection in Perpetuity	859.4	836.6	804.8	771.7	736.7
		Injection in Perpetuity with Replanting	859.4	836.6	804.8	771.7	736.7
	**Non-Ash Trees**	Remove Dead Ash Only	0.0	0.0	0.0	0.0	0.0
		Remove Dead Ash then Replant	0.0	28.9	59.9	96.8	141.1
		Preemptive Removal then Replant	12.0	52.3	83.9	123.5	168.7
		Replant, Inject, then Preemptive Removal	12.0	61.4	106.0	160.5	224.0
		Inject, Preemptive Removal, and Replant	1.2	6.8	57.3	91.4	133.5
		Injection in Perpetuity	0.0	0.0	0.0	0.0	0.0
		Injection in Perpetuity with Replanting	1.2	6.8	17.0	31.4	50.6
	**Total Trees**	Remove Dead Ash Only	707.0	316.6	115.9	43.4	17.3
		Remove Dead Ash then Replant	707.0	345.4	175.8	140.1	158.4
		Preemptive Removal then Replant	658.5	52.3	83.9	123.5	168.7
		Replant, Inject, then Preemptive Removal	871.4	898.0	106.0	160.5	224.0
		Inject, Preemptive Removal, and Replant	860.6	843.4	57.3	91.4	133.5
		Injection in Perpetuity	859.4	836.6	804.8	771.7	736.7
		Injection in Perpetuity with Replanting	860.6	843.4	821.8	803.0	787.3

**Fig. 2.**
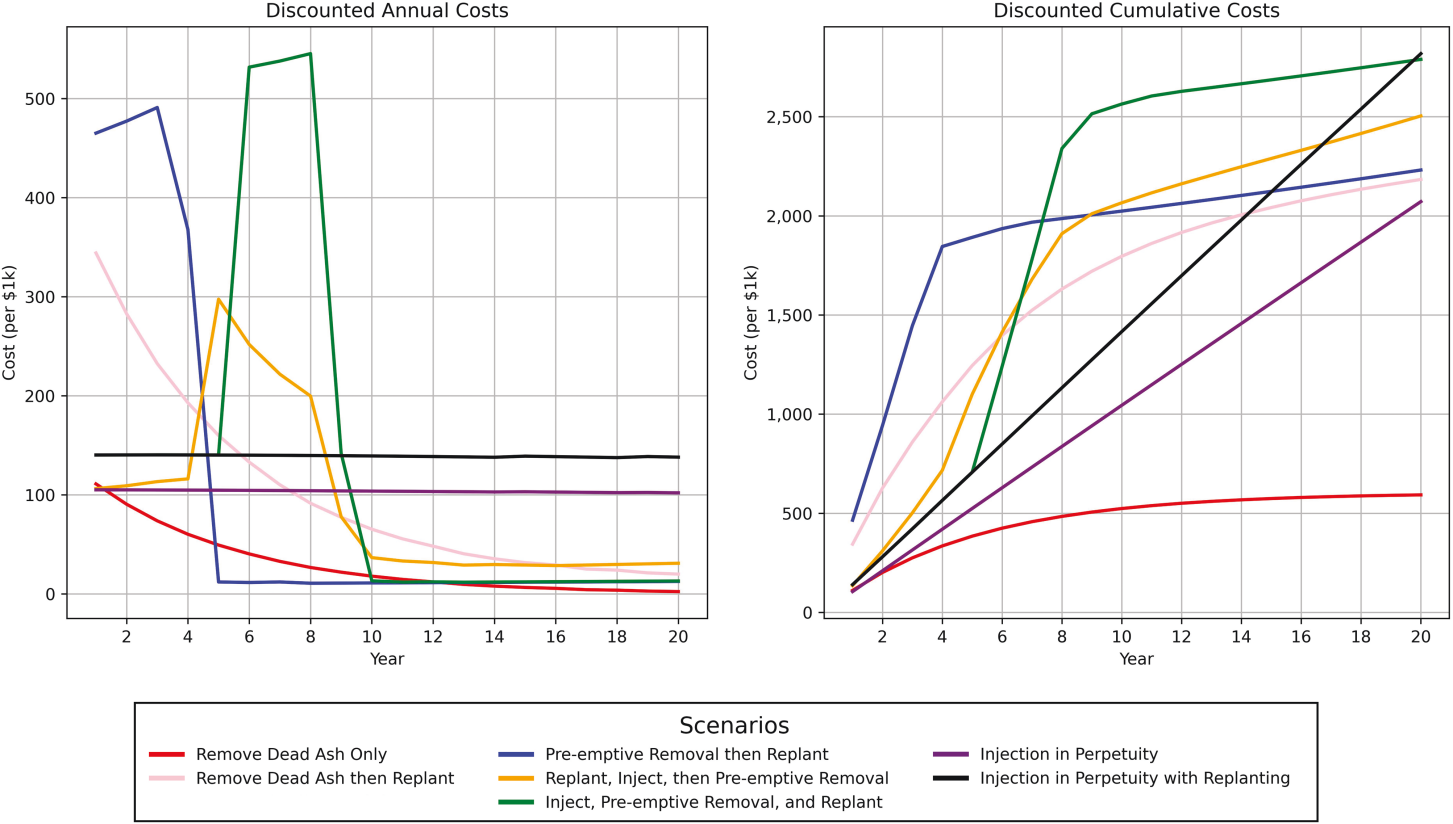
Annual and cumulative costs of 7 EAB management scenarios, discounted at a rate of 2.83% per annum.

**Fig. 3.**
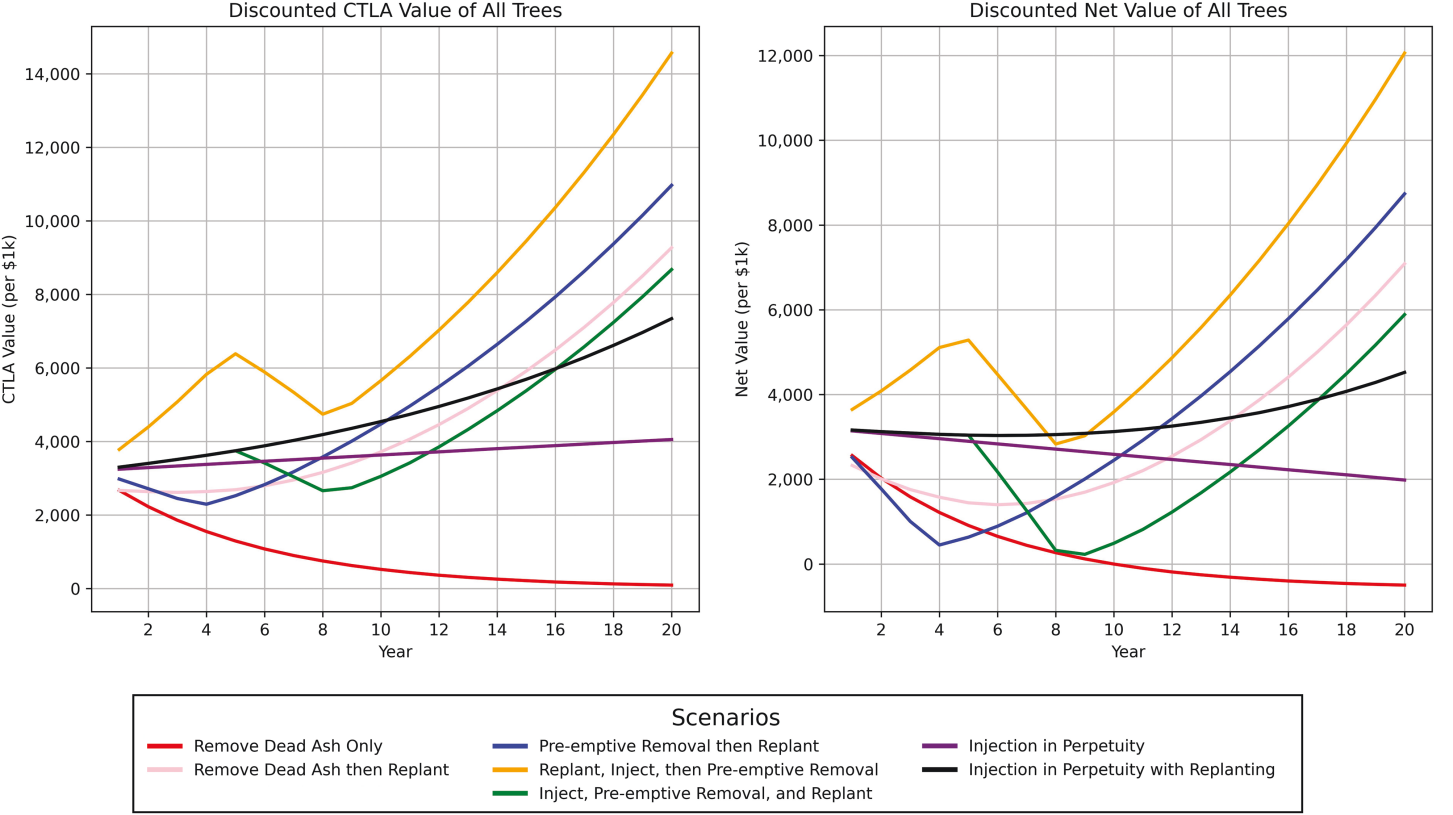
Discounted value of ash and non-ash trees calculated using the Council of Tree and Landscape Appraisers (CTLA) (2019) trunk formula technique and the discounted net value, calculated by deducting annual cost from the CTLA value.

Cumulative costs were greatest under the Replant, Inject, then Preemptive Removal option (Fig. 2) (Table 6), although this option had the greatest discounted CTLA value and discounted net value at Year 20 (Fig. 3). Besides the Remove Only option, the least expensive was Injection in Perpetuity. Only the Remove Only simulation had discounted cumulative costs that exceeded the discounted CTLA value of the trees, resulting in a net loss.

**Table 6.** Comparison of discounted cost and value of 7 emerald ash borer management options at Years 1, 5, 10, 15, and 20 based on 2024 management rates with a 2.83% discount rate.

Metric	Scenario	Year 1	Year 5	Year 10	Year 15	Year 20
**Inflation-Adjusted** **Cumulative Cost** (per $1000 CAD)	Remove Dead Ash Only	111.0	343.9	407.5	388.5	348.7
	Remove Dead Ash then Replant	344.2	1,113.4	1,397.2	1,381.5	1,285.0
	Preemptive Removal then Replant	465.1	1,691.7	1,574.4	1,436.7	1,313.0
	Replant, Inject, then Preemptive Removal	135.2	983.9	1607.1	1,548.9	1,473.4
	Inject, Preemptive Removal, and Replant	140.2	632.3	1,994.5	1,817.3	1,641.3
	Injection in Perpetuity	105.0	468.7	812.5	1,055.6	1,219.2
	Injection in Perpetuity with Replanting	140.2	632.3	1,101.6	1,434.9	1,658.4
**Inflation-Adjusted CTLA Value of All Trees** (per $1000 CAD)	Remove Dead Ash Only	2,670.5	1,157.6	407.0	146.2	56.0
	Remove Dead Ash then Replant	2,670.5	2,405.5	2,892.8	4,001.1	5,454.9
	Preemptive Removal then Replant	2,978.9	2,259.0	3,479.1	4,918.0	6,453.9
	Replant, Inject, then Preemptive Removal	3,783.2	5,710.8	4,399.1	6,394.6	8,569.5
	Inject, Preemptive Removal, and Replant	3,302.0	3,352.3	2,375.3	3,640.4	5,105.5
	Injection in Perpetuity	3,246.3	3,059.4	2,826.1	2,602.3	2,385.6
	Injection in Perpetuity with Replanting	3,302.0	3,352.3	3,533.1	3,851.2	4,319.9
**Inflation-Adjusted** **Net Value of All Trees** (per $1000 CAD)	Remove Dead Ash Only	2,559.5	813.7	−0.5	−242.3	−292.7
	Remove Dead Ash then Replant	2,326.3	1,292.0	1,495.6	2,619.6	4,169.9
	Preemptive Removal then Replant	2,513.8	567.3	1,904.7	3,481.3	5,140.9
	Replant, Inject, then Preemptive Removal	3,648.0	4,726.9	2,792.0	4,845.7	7,096.1
	Inject, Preemptive Removal, and Replant	3,161.8	2,719.9	380.8	1,823.2	3,464.2
	Injection in Perpetuity	3,141.2	2,590.6	2,013.6	1,546.7	1,166.4
	Injection in Perpetuity with Replanting	3,161.8	2,719.9	2,431.5	2,416.2	2,661.5

### Normalized Cost Comparison

**Fig. 4.**
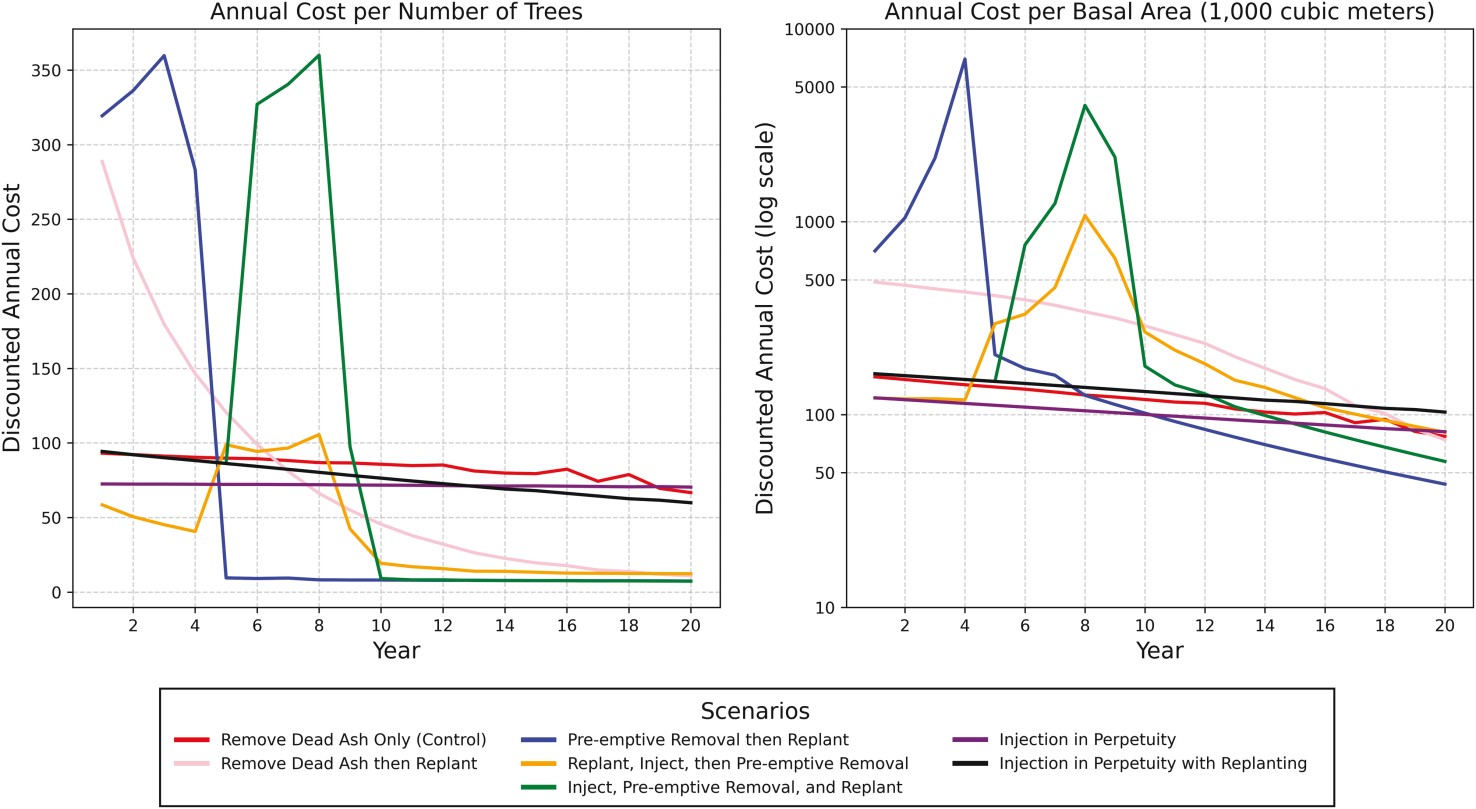
Discounted annual costs per number of ash and non-ash trees and their basal area per 1,000 m^3^, calculated using diameter at breast height. Note that the basal area graph uses a log scale.

In examining the discounted annual cost per tree (Fig. 4), both the Preemptive Removal then Replant option and Inject, Preemptive Removal, and Replant had substantial spikes. In Year 3, the Preemptive Removal then Replant option reached a maximum annual cost per tree of $359.70 while in Year 8, the Inject, Preemptive Removal, and Replant option reached a maximum cost per tree of $360.00. By Year 10, these two management options became the least expensive on a per annum basis. Similarly, these two options had spikes in Year 4 and Year 8, respectively, when discounted annual cost is examined per 1,000 m^3^ of basal area (Fig. 4); however, the Preemptive Removal then Replant was the least expensive of all options from Year 5 onwards. In examining discounted annual cost both per tree and per basal area, the selection of options based on annual cost minimization in the first 10 yr can lead to higher annual costs in the decade following, a trend observable in examining the overall discounted annual costs and, to a lesser extent, the discounted cumulative costs (Fig. 2).

**Fig. 5.**
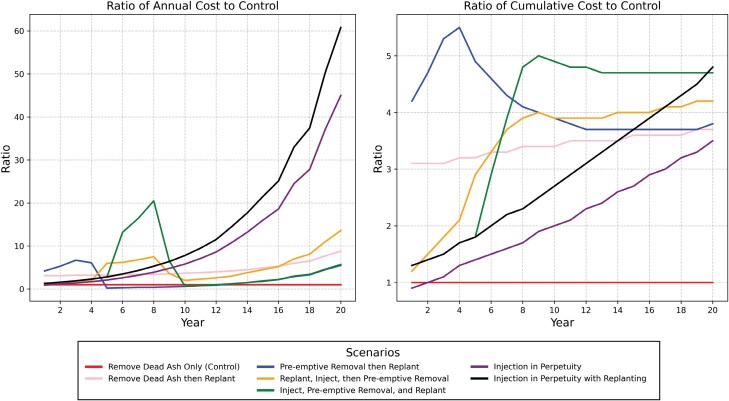
Tradeoff ratios comparing the discounted annual and cumulative cost of 6 EAB management scenarios to a control option of removing ash as they become infested and die from EAB with no replanting.

In considering the ratio of discounted annual cost to the control option—Remove Dead Ash Only—both injection in perpetuity options resulted in a cost ratio as high as 60.8 times the control and more than 11 times the option with the small ratio, Preemptive Removal then Replant (Fig. 5). The two injection in perpetuity options showed an accelerating cost ratio when compared to the control, which will build past Year 20. A similar pattern was observed in the ratio of the cumulative cost to the control option of Remove Dead Ash Only. While the Preemptive Removal then Replant and Inject, Preemptive Removal, and Replant options spiked before decreasing, the injection in perpetuity options continued to grow.

### Effects of Management Rates

In optimizing 4 management decisions—(i) the year that injections end, (ii) the year that removals start, (iii) the rate of tree removals per year, and (iv) the number of new trees planted per year—there were changes to 3 management options: Preemptive Removal then Replant (iii); Replant, Inject, the Preemptive Removal (ii, iii, & iv); and Inject, Preemptive Removal, and Replant (i & iii).

In the Preemptive Removal then Replant simulation, the greatest number of trees in Year 20 occurred with the lowest annual rate of tree removals (100 trees per year). The low annual rate of tree removals also had the smallest spike in discounted annual costs. However, basal area, discounted CTLA value, and discounted net value at Year 20 were greatest with the highest annual rate of tree removals (1,000 trees per year). Similarly, the discounted cumulative cost was lowest with the highest annual rate of tree removals.

In the Inject, Preemptive Removal, and Replant, the greatest number of trees and greatest basal area in Year 20 occur with the lowest annual rate of tree removals (100 trees per year) and the latest end to tree injections (Year 20). However, discounted CTLA value and discounted net value at Year 20 were greatest with the highest annual rate of tree removals (1,000 trees per year) and the earliest end to tree injections (Year 1). Similarly, the discounted cumulative cost was lowest with the highest annual rate of tree removals and earliest end to tree injections.

In the Replant, Inject, then Preemptive Removal, all 4 management decisions influenced the outcome of the simulation. Total tree count and total basal area benefited most from the latest start to removals (Year 20), the lowest removal rate (100 trees/year), and beginning proactive replanting in Year 1. Total tree count benefited most from a planting rate of 333 trees per year versus 1,000 trees per year for total basal area. Discounted CTLA value and discounted net value both benefited from the latest start to removals (Year 20), the lowest removal rate (100 trees/year), and the highest proactive replanting rate (1,000 trees/year) beginning in Year 1. Discounted cumulative costs benefited most from the highest annual removal rate (1,000 trees/year), the lowest planting rate (100 trees/year), beginning to plant in Year 1, and a start to preemptive removals as early as possible (Year 2 based on our proactive replanting rules).

## Discussion

Our results highlight that the best method for EAB management depends on the underlying objectives. This is a new framing of bioeconomic analyses for EAB management as previous studies have made conclusions and recommendations based on one objective. This is also the first study that we know of to evaluate management options for late-stage EAB infestations following successive years of ash loss. Our model and its results for the Mississauga case study identify several important options. Here, we provide recommendations based on common urban forestry objectives. We conclude with limitations of our model and recommendations for future research directions.

### Objective: Preserve the Ash Population

Preserving the ash genus has been suggested as an important consideration for EAB management. For urban foresters managing for this objective, continuing long-term ash injections is the most appropriate option, although it also comes with the highest annual costs after Year 10 (Fig. 2). Secondary objectives like cost minimization or basal area maximization will decide whether dying ash trees should be replaced after they are deemed unsuitable for continued injections. Based on the dendrochronological data and the Mississauga ash tree inventory, injected ash trees have reduced growth and tree condition continues to depreciate. This contrasts with previous studies that have assumed tree condition and growth rate do not change with injections. Although arboricultural best practices for basal injections can improve the tree’s ability to compartmentalize the drill holes, Mississauga’s injected ash trees have higher annual mortality rates than other species. This is observed in Fig. 1 where only 852 ash trees remain at Year 20, 57.18% of the ash population in Year 0. Thus, the long-term preservation of the ash population using injections is unlikely due to their continued mortality.

### Objective: Maximizing the Number of Trees

Many urban foresters set urban forestry targets based on the number of trees, including the number of trees planted and the number of trees retained. For cities shifting from large street trees like ash and elm (*Ulmus* spp.) to smaller stature genera like American linden (*Tilia americana* L.) or *Prunus* spp., this may be a more appropriate objective than preserving basal area. When considering EAB management that will ensure that the number of trees (stem count) is retained, Replant, Inject, then Preemptive Removal is the best performing management objective as it replants trees while maintaining ash trees through injections while the replacement trees establish. This maintains a relatively consistent total tree count over the 20-yr period. While it incurs the second highest annual cost maximum—only slightly less than Inject, Preemptive Removal and Replant and near-identical maxima when comparing the annual cost per tree—the discounted cumulative cost is a near-median value from Year 9 onwards. The planting of trees in alternative locations was not considered by previous models, which explains why they found continued injections to be the most beneficial option. Across the management objectives, the gradual decline in tree count is due to the mortality of newly planted trees.

### Objective: Maximizing the Basal Area

Many commonly cited ecosystem services are maximized in larger trees, including carbon sequestration and storage and capture of stormwater and air pollution (Hand et al. 2019). This objective prefers management options that will ensure basal area is maximized. Both injection options vastly outperform the other management options but come with high costs from Year 10 onwards and a linear increase in discounted cumulative costs. Adding replanting to the injection program results in a greater basal area in later years, but at the highest annual cost after Year 9 and the highest discounted cumulative cost at Year 20. The underperformance of the other management options reflects the removal of large, mature ash trees, resulting in a loss of basal area that will take several decades to reestablish.

### Objective: Long-term Cost Reduction

For urban forestry departments with limited budgets, cost reduction is an important consideration. Similarly, cities like Mississauga have reached the end of their leveraged EAB management funds that were used to support the initial municipal response to EAB. This objective provides important insight into minimizing net cost for EAB management. This objective prefers management options that will result in the lowest net cost over a 20-year period. In considering cost reduction, the lowest cost is achieved through the Remove Dead Ash Only option, which ceases injection programs and removes ash trees as they die. The mortality of non-injected ash has previously been estimated to be 20% per year. For cities able to sustain this many removals, removing ash as they die is magnitudes less costly than other management objectives, including up to 60 times less costly than Injection in Perpetuity with Replanting within the 20-yr period. While continued injections maintain an increasing and costly trajectory, cities nearing the end of their EAB management funds should consider preemptive removal and replanting or replanting non-injected ash as they are removed, which both result in high costs incurred in the short-term but low annual costs quickly thereafter and help restore the urban forest tree count and basal area.

### Objective: Lowest Annual Costs

A related objective to long-term cost reduction is to minimize the annual costs. Here, management options are considered more preferable when they avoid spikes in annual costs. While Remove Dead Ash Only (Control) has the lowest annual costs, it is close to both injection programs, with replanting adding a small additional cost to injection programs. Where injections are ill-preferred because of their high cumulative costs, ceasing injections and removing ash trees as they die and replanting yields the next lowest annual cost while still planting replacement trees. From Year 6 onwards, this option is also less expensive annually than injections. All preemptive removal options have high annual costs, although these can be minimized by removing fewer ash trees per year or planting fewer replacement trees.

### Objective: Value Maximization

Considering the net value of all trees incorporates tree count, basal area, costs, and CTLA value. Differing from previous studies, we found that proactive planting while injecting ash before their preemptive removal results in the greatest net value in the short and long terms. This is followed by preemptive removal and replanting, which allows trees to be planted in the same location as the removed ash. For cities that have little remaining street or park space for tree planting, the latter may be the only option if alternative planting sites are not available to facilitate the former management option. This was the approach used by Vannatta et al. (2012) who only applied tree planting retroactively. Interestingly, the two continued injection programs had a low net value, being outcompeted in both the short and long term. Injections in perpetuity without replanting result in a decreasing net value, the only decrease besides the Remove Dead Ash Only option. This finding, differing from that of Vannatta et al. (2012), is predominantly a reflection of our depreciation of the ash genus in the CTLA equation, in keeping with industry appraisal recommendations from the Council of Tree & Landscape Appraisers. (2019).

### Limitations and Future Research Directions

In this study, we generalized the ash tree inventory akin to Vannatta et al. (2012), which makes it easier for the model to be repurposed for other urban forests. This also decreases the computational demand. An individualized analysis that includes probabilistic frameworks increases complexity and requires spatially explicit modeling, which is likely inaccessible for urban foresters based on the requirements of an individualized, probabilistic Dutch elm disease (*Ophiostoma* spp.) model (Bajeux et al. 2020). As a result, urban foresters will have to establish a matrix for deciding what trees in their communities should be removed versus injected with TreeAzin. Previous guidance has suggested that trees in good to excellent condition should be prioritized for injection, and that canopy thinning > 60% should make a tree ineligible for injection (Smitley et al. 2010). Similarly, ash trees that cause conflict with infrastructure or provide limited benefits could be replaced with new, alternative species that better fit the site.

Three important areas of future research involve determining ash mortality rates. Firstly, future studies should test our injected ash mortality rates in other municipalities to evaluate variance in ash mortality during a prolonged injection program. Secondly, longitudinal studies are required to determine condition rating decline in injected ash after multiple injections. For the purposes of this study, we used a consistent mortality rate and condition rating, but if injected ash are worsening each year, the benefits of an injection program would be increasingly diminished. Thirdly, the mortality rate of uninjected ash must be quantified for late-stage EAB infestation. If mortality rates of uninjected ash are lower than the 20% used by Vannatta et al. (2012), then the value of ash will be sustained longer and the annual costs will be diminished.

As ash trees are lost from the urban environment, it is also imperative that researchers foster relationships with urban forestry departments. Studies on ash growth rates, compartmentalization, and condition decline will all benefit bioeconomic modeling. These partnerships are equally important to ensure knowledge translation from academic researchers to a largely practitioner audience.

## Supplementary material

Supplementary material is available at *Journal of Economic Entomology* online.

toaf037_suppl_Supplementary_Material
