# Supplementary material for: A bioeconomic analysis of objective-based management options for late-stage emerald ash borer (Coleoptera: Buprestidae) infestations
Source: J Econ Entomol. 2025 Apr 4;118(3):1307–19. doi: 10.1093/jee/toaf037 (PMC12167853; doi:10.1093/jee/toaf037)
Supplement: toaf037_suppl_Supplementary_Material [file toaf037_suppl_supplementary_material.docx]

**Supplementary Material: Future Value and Costs without Discounting**

In this supplementary material, we provide figures and tables with an inflation rate of 2% per annum to estimate the future value of the tree, the midpoint of the inflation-control target of the Bank of Canada (2024). McKenney et al. (2012) and Brukas et al. (2001) both recommend reporting future values with a zero discount rate to show the impact of discounting, which we provide here as both figures and tables. The findings reported in the Results for present value remain unchanged as the relationship between the scenarios is preserved in the absence of discounting.

**Figures**

***Tree Count and Basal Area***


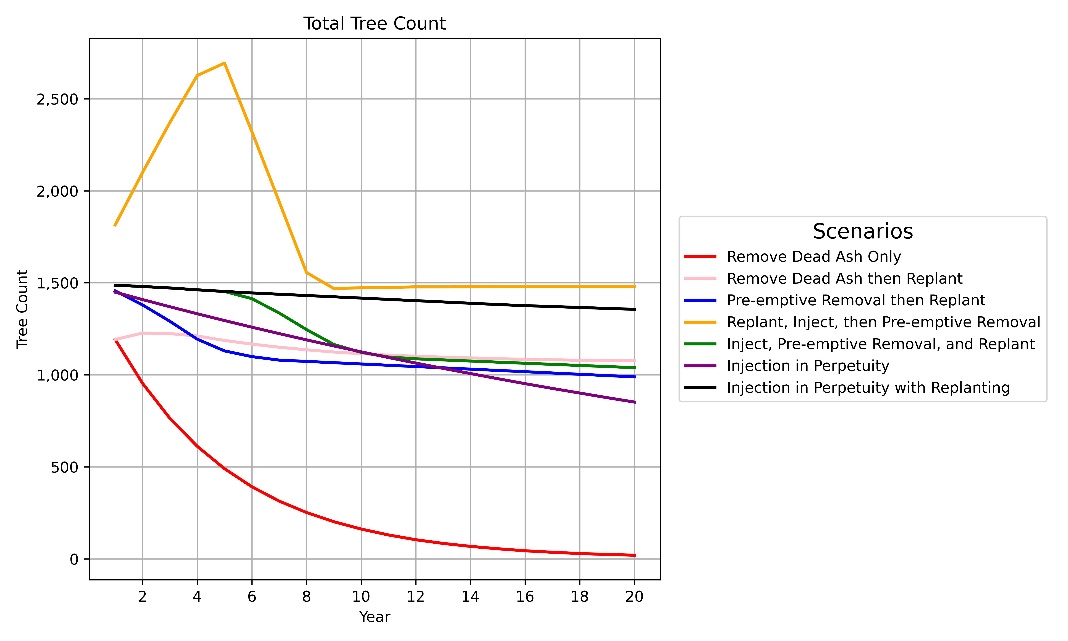


Figure S.1: Number of ash (*Fraxinus* spp.) and non-ash replacement trees under seven emerald ash borer (EAB) (*Agrilus planipennis*) management scenarios between Year 1 and Year 20.


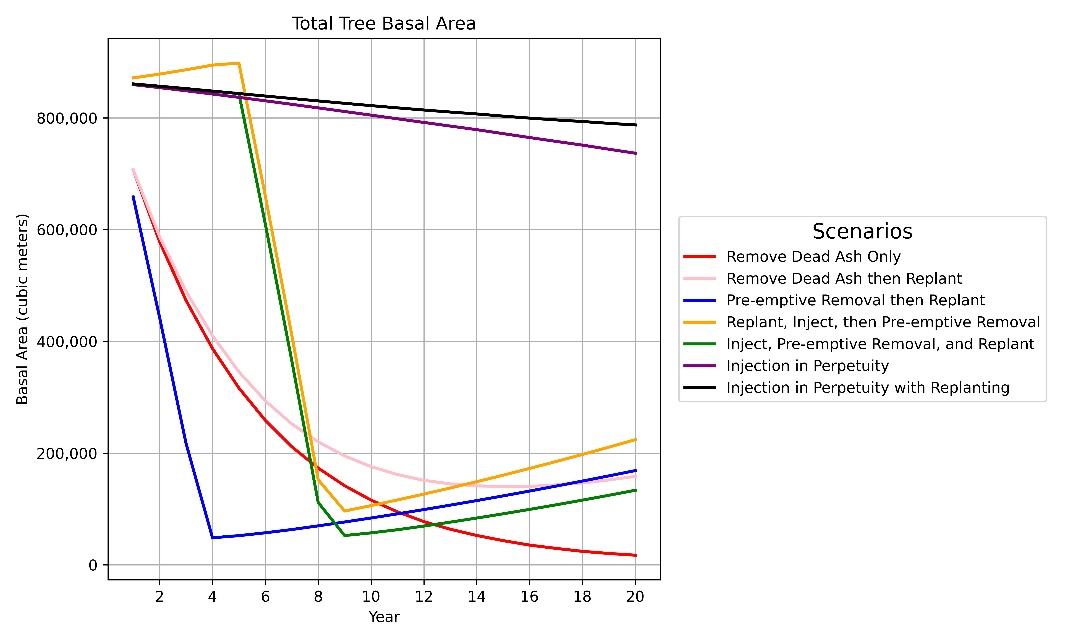


Figure S.2: Basal area, calculated from diameter at breast height (DBH), for both ash and non-ash replacement trees under seven EAB management scenarios between Year 1 and Year 20.

***Annual and Cumulative Costs with Inflation***

**
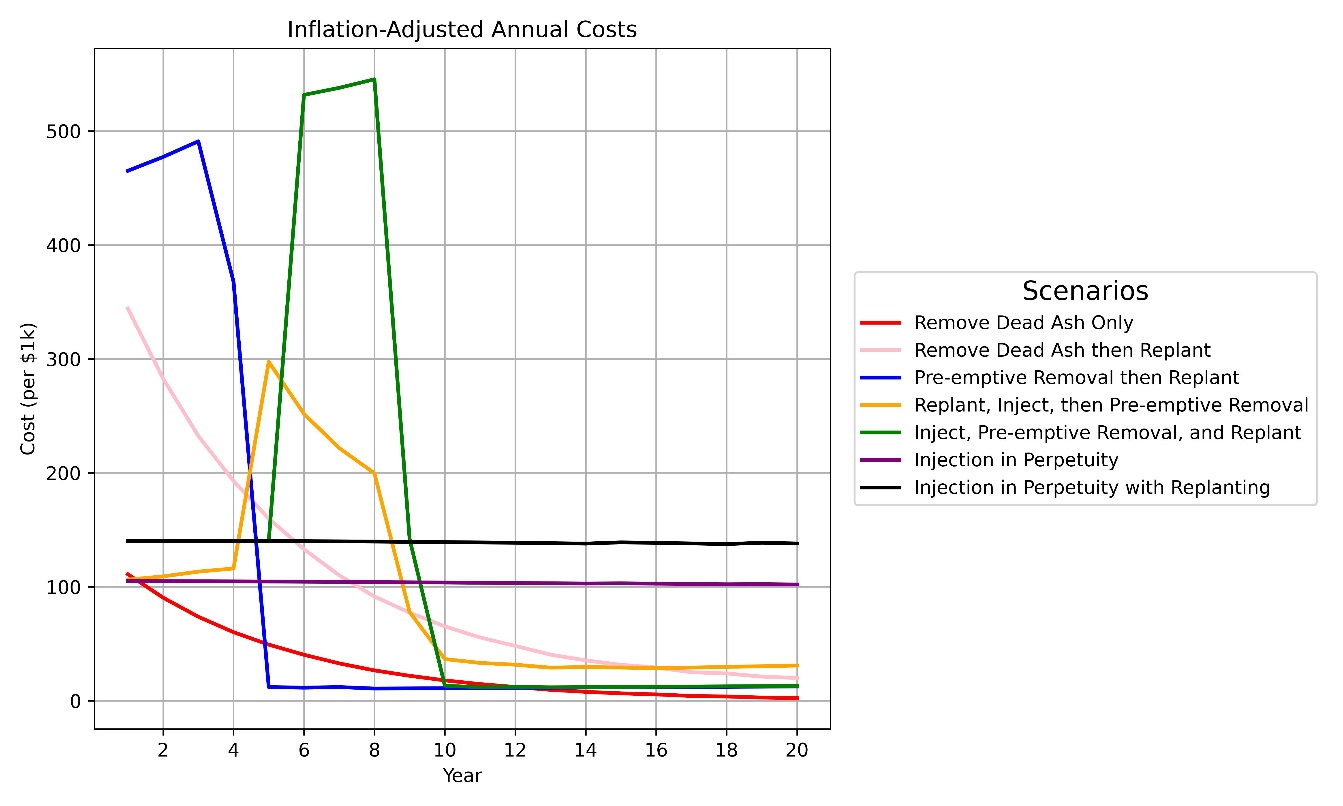
**

Figure S.3: Annual costs of various EAB management scenarios between Year 1 and Year 20, adjusted for inflation at 2% per annum.

**
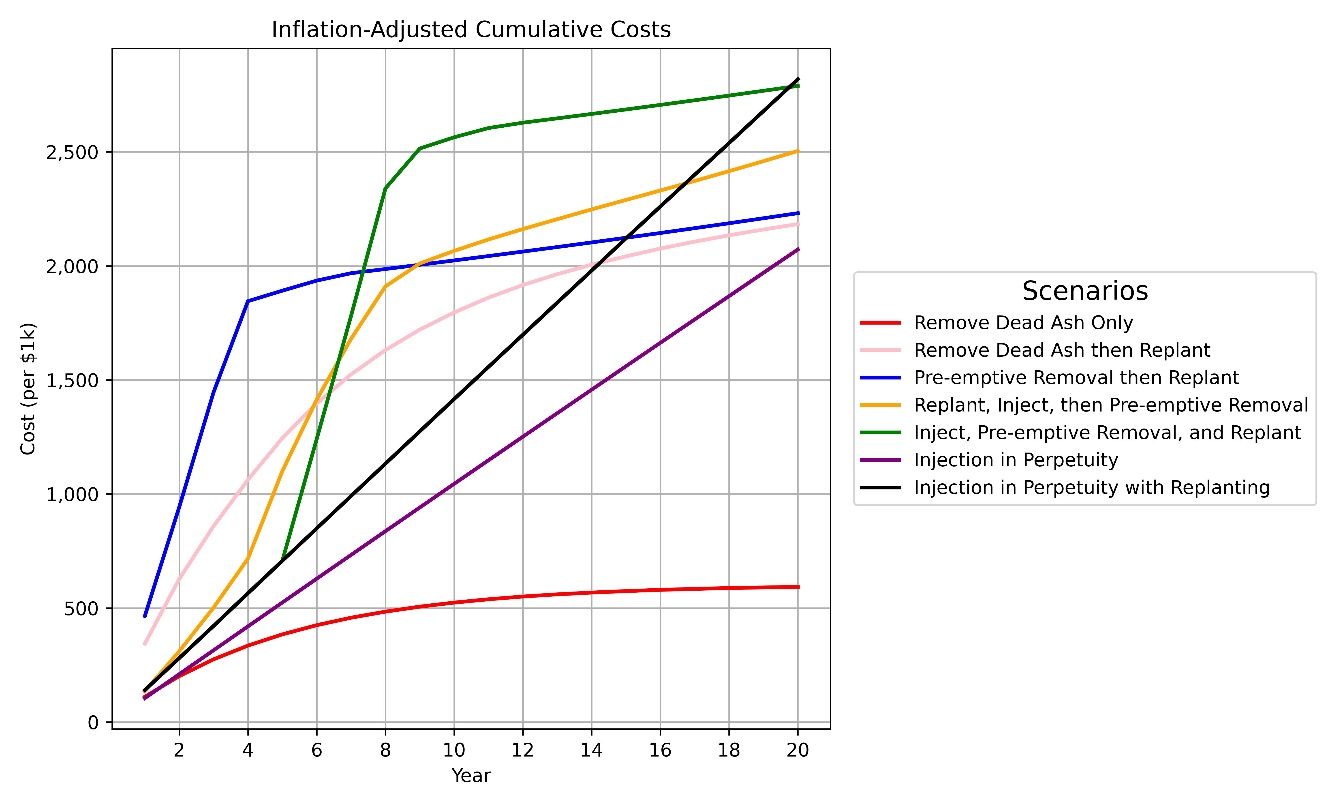
**

Figure S.4: Cumulative costs of various EAB management scenarios between Year 1 and Year 20, adjusted for inflation at 2% per annum.

***CTLA Value and Net Value with Inflation***

**
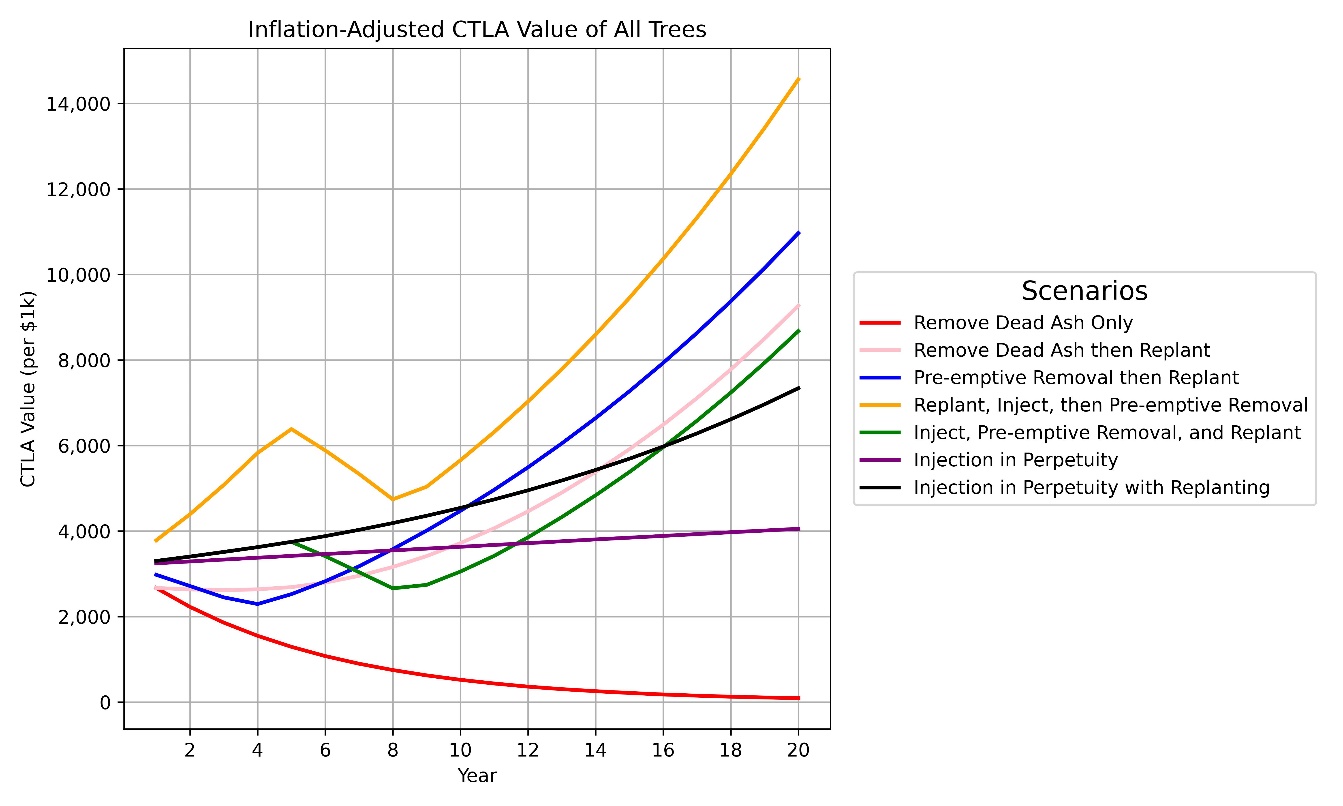
**

Figure S.5: Council of Tree and Landscape Appraisers (2019) (CTLA) trunk formula technique-based valuation of ash and non-ash under various EAB management scenarios between Year 1 and Year 20, adjusted for inflation at 2% per annum.

**
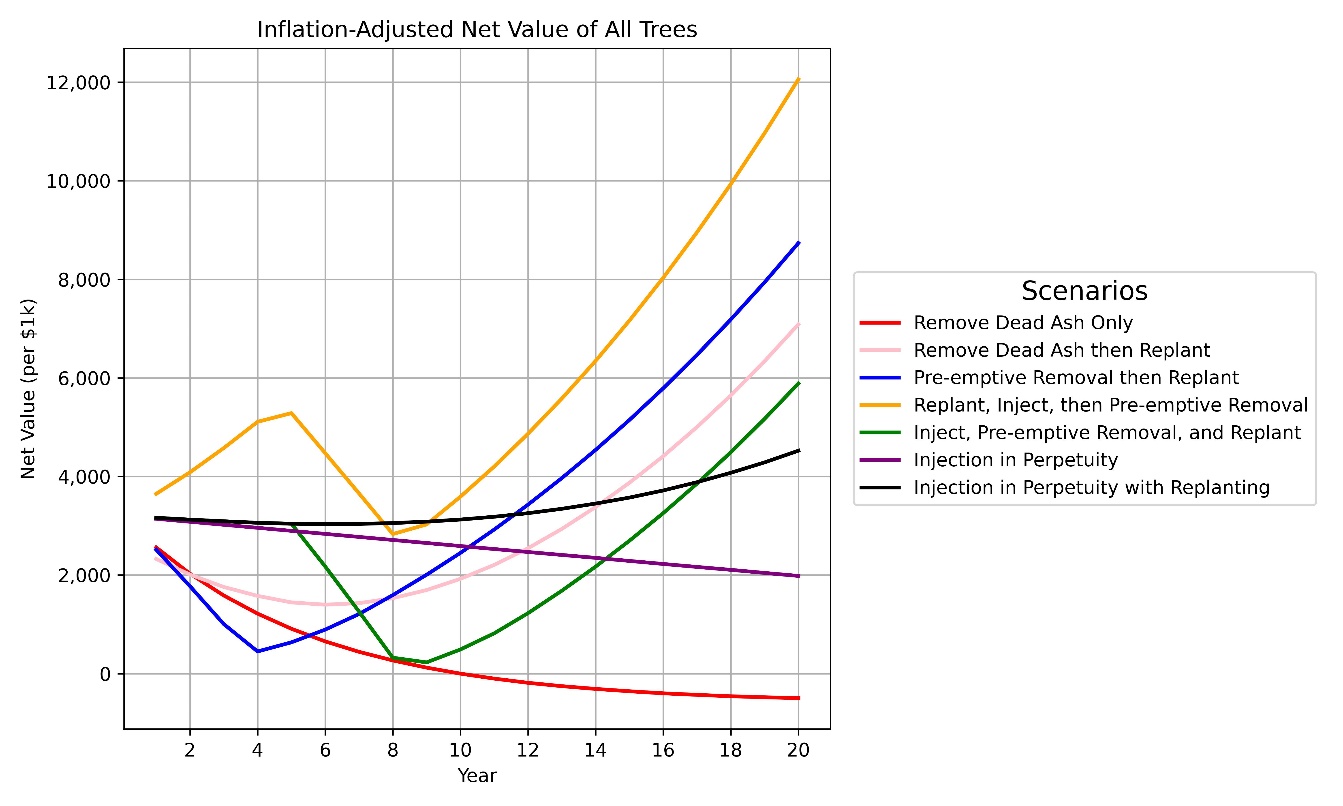
**

Figure S.5: CTLA value minus management costs under various EAB management scenarios between Year 1 and Year 20, adjusted for inflation at 2% per annum.

**Tables**

Table S.1: Tree biometric variables associated with seven emerald ash borer management scenarios.

| **Metric** | **Tree Species** | **Scenario** | **Year 1** | **Year 5** | **Year 10** | **Year 15** | **Year 20** |
| --- | --- | --- | --- | --- | --- | --- | --- |
| **Tree Count** | **Ash Trees** | Remove Dead Ash Only | 1,192 | 490 | 162 | 55 | 20 |
|  |  | Remove Dead Ash then Replant | 1,192 | 490 | 162 | 55 | 20 |
|  |  | Pre-emptive Removal then Replant | 1,090 | 0 | 0 | 0 | 0 |
|  |  | Replant, Inject, then Pre-emptive Removal | 1,449 | 1,295 | 0 | 0 | 0 |
|  |  | Inject, Pre-emptive Removal, and Replant | 1,449 | 1,295 | 0 | 0 | 0 |
|  |  | Injection in Perpetuity | 1,449 | 1,295 | 1,125 | 979 | 852 |
|  |  | Injection in Perpetuity with Replanting | 1,449 | 1,295 | 1,125 | 979 | 852 |
|  | **Non-Ash Trees** | Remove Dead Ash Only | 0 | 0 | 0 | 0 | 0 |
|  |  | Remove Dead Ash then Replant | 0 | 697 | 953 | 1,033 | 1,056 |
|  |  | Pre-emptive Removal then Replant | 366 | 1,130 | 1,059 | 1,024 | 989 |
|  |  | Replant, Inject, then Pre-emptive Removal | 366 | 1,399 | 1,473 | 1,480 | 1,480 |
|  |  | Inject, Pre-emptive Removal, and Replant | 38 | 158 | 1,122 | 1,069 | 1,039 |
|  |  | Injection in Perpetuity | 0 | 0 | 0 | 0 | 0 |
|  |  | Injection in Perpetuity with Replanting | 38 | 158 | 292 | 403 | 504 |
|  | **All Trees** | Remove Dead Ash Only | 1,192 | 490 | 162 | 55 | 20 |
|  |  | Remove Dead Ash then Replant | 1,192 | 1,187 | 1,115 | 1,088 | 1,076 |
|  |  | Pre-emptive Removal then Replant | 1,456 | 1,130 | 1,059 | 1,024 | 989 |
|  |  | Replant, Inject, then Pre-emptive Removal | 1,815 | 2,694 | 1,473 | 1,480 | 1,480 |
|  |  | Inject, Pre-emptive Removal, and Replant | 1,487 | 1,453 | 1,122 | 1,069 | 1,039 |
|  |  | Injection in Perpetuity | 1,449 | 1,295 | 1,125 | 979 | 852 |
|  |  | Injection in Perpetuity with Replanting | 1,487 | 1,453 | 1,417 | 1,382 | 1,356 |
| **Basal Area (m^3^)** | **Ash Trees** | Remove Dead Ash Only | 706,967 | 316,551 | 115,889 | 43,353 | 17,293 |
|  |  | Remove Dead Ash then Replant | 706,967 | 316,551 | 115,889 | 43,353 | 17,293 |
|  |  | Pre-emptive Removal then Replant | 646,472 | 0 | 0 | 0 | 0 |
|  |  | Replant, Inject, then Pre-emptive Removal | 859,392 | 836,600 | 0 | 0 | 0 |
|  |  | Inject, Pre-emptive Removal, and Replant | 859,392 | 836,600 | 0 | 0 | 0 |
|  |  | Injection in Perpetuity | 859,392 | 836,600 | 804,786 | 771,690 | 736,685 |
|  |  | Injection in Perpetuity with Replanting | 859,392 | 836,600 | 804,786 | 771,690 | 736,685 |
|  | **Non-Ash Trees** | Remove Dead Ash Only | 0 | 0 | 0 | 0 | 0 |
|  |  | Remove Dead Ash then Replant | 0 | 28,887 | 59,925 | 96,774 | 141,139 |
|  |  | Pre-emptive Removal then Replant | 12,033 | 52,295 | 83,870 | 123,461 | 168,718 |
|  |  | Replant, Inject, then Pre-emptive Removal | 12,033 | 61,380 | 106,049 | 160,531 | 224,025 |
|  |  | Inject, Pre-emptive Removal, and Replant | 1,249 | 6,780 | 57,260 | 91,389 | 133,468 |
|  |  | Injection in Perpetuity | 0 | 0 | 0 | 0 | 0 |
|  |  | Injection in Perpetuity with Replanting | 1,249 | 6,780 | 17,042 | 31,351 | 50,566 |
|  | **All Trees** | Remove Dead Ash Only | 706,967 | 316,551 | 115,889 | 43,353 | 17,293 |
|  |  | Remove Dead Ash then Replant | 706,967 | 345,439 | 175,814 | 140,127 | 158,432 |
|  |  | Pre-emptive Removal then Replant | 658,505 | 52,295 | 83,870 | 123,461 | 168,718 |
|  |  | Replant, Inject, then Pre-emptive Removal | 871,426 | 897,981 | 106,049 | 160,531 | 224,025 |
|  |  | Inject, Pre-emptive Removal, and Replant | 860,642 | 843,381 | 57,260 | 91,389 | 133,468 |
|  |  | Injection in Perpetuity | 859,392 | 836,600 | 804,786 | 771,690 | 736,685 |
|  |  | Injection in Perpetuity with Replanting | 860,642 | 843,381 | 821,829 | 803,041 | 787,252 |

Table S.2: Inflation-adjusted costs and values associated with seven emerald ash borer management scenarios in thousands of Canadian dollars. An inflation of 2% is used per Bank of Canada (2024).

| **Metric** | **EAB Management Scenario** | **Year 1** | **Year 5** | **Year 10** | **Year 15** | **Year 20** |
| --- | --- | --- | --- | --- | --- | --- |
| **Inflation-Adjusted**  **Cumulative Cost**  (per $1,000 CAD) | Remove Dead Ash Only | 111.0 | 384.5 | 523.8 | 574.2 | 592.6 |
|  | Remove Dead Ash then Replant | 344.2 | 1,244.9 | 1,796.1 | 2,041.9 | 2,183.7 |
|  | Pre-emptive Removal then Replant | 465.1 | 1,891.4 | 2,024.0 | 2,123.4 | 2,231.2 |
|  | Replant, Inject, then Pre-emptive Removal | 135.2 | 1,100.1 | 2,065.9 | 2,289.3 | 2,503.9 |
|  | Inject, Pre-emptive Removal, and Replant | 140.2 | 707.0 | 2,563.9 | 2,685.9 | 2,789.1 |
|  | Injection in Perpetuity | 105.0 | 524.1 | 1,044.5 | 1,560.2 | 2,071.9 |
|  | Injection in Perpetuity with Replanting | 140.2 | 707.0 | 1,416.1 | 2,120.9 | 2,818.2 |
| **Inflation-Adjusted CTLA Value of All Trees**  (per $1,000 CAD) | Remove Dead Ash Only | 2,670.5 | 1,294.3 | 523.2 | 216.1 | 95.2 |
|  | Remove Dead Ash then Replant | 2,670.5 | 2,689.5 | 3,718.7 | 5,913.7 | 9,269.7 |
|  | Pre-emptive Removal then Replant | 2,978.9 | 2,525.8 | 4,472.4 | 7,268.9 | 10,967.3 |
|  | Replant, Inject, then Pre-emptive Removal | 3,783.2 | 6,385.3 | 5,655.1 | 9,451.3 | 14,562.4 |
|  | Inject, Pre-emptive Removal, and Replant | 3,302.0 | 3,748.2 | 3,053.4 | 5,380.6 | 8,675.9 |
|  | Injection in Perpetuity | 3,246.3 | 3,420.7 | 3,633.1 | 3,846.2 | 4,053.9 |
|  | Injection in Perpetuity with Replanting | 3,302.0 | 3,748.2 | 4,541.9 | 5,692.0 | 7,340.9 |
| **Inflation-Adjusted**  **Net Value of All Trees**  (per $1,000 CAD) | Remove Dead Ash Only | 2,559.5 | 909.8 | -0.7 | -358.1 | -497.5 |
|  | Remove Dead Ash then Replant | 2,326.3 | 1,444.6 | 1,922.6 | 3,871.8 | 7,086.0 |
|  | Pre-emptive Removal then Replant | 2,513.8 | 634.3 | 2,448.5 | 5,145.4 | 8,736.1 |
|  | Replant, Inject, then Pre-emptive Removal | 3,648.0 | 5,285.1 | 3,589.2 | 7,162.0 | 12,058.5 |
|  | Inject, Pre-emptive Removal, and Replant | 3,161.8 | 3,041.2 | 489.5 | 2,694.7 | 5,886.7 |
|  | Injection in Perpetuity | 3,141.2 | 2,896.6 | 2,588.6 | 2,286.0 | 1,982.0 |
|  | Injection in Perpetuity with Replanting | 3,161.8 | 3,041.2 | 3125.7 | 3,571.2 | 4,522.7 |

**References for Supplementary Information**

Bank of Canada. (2024). *Inflation*. Bank of Canada. Retrieved November 10, 2024 from <https://www.bankofcanada.ca/core-functions/monetary-policy/inflation/>

Brukas, V., Jellesmark Thorsen, B., Helles, F., & Tarp, P. (2001). Discount rate and harvest policy: implications for Baltic forestry. *Forest Policy and Economics*, *2*(2), 143-156. <https://doi.org/https://doi.org/10.1016/S1389-9341(01)00050-8>

Council of Tree & Landscape Appraisers. (2019). *Guide for Plant Appraisal* (10 ed.). International Society of Arboriculture.

McKenney, D. W., Pedlar, J. H., Yemshanov, D., Lyons, B., Campbell, K. L., & Lawrence, K. (2012). Estimates of the potential cost of emerald ash borer (*Agrilus planipennis* Fairmaire) in Canadian municipalities. *Arboriculture & Urban Forestry*, *38*(3), 81-89.
